# A holistic approach to eye care for older people

**Published:** 2008-06

**Authors:** AB Dey, Robert Lindfield, Ashish Goel

**Affiliations:** Professor, Department of Medicine & Chief of Geriatric Services, All India Institute of Medical Sciences, New Delhi 110 029, India.; Honorary Lecturer, International Centre for Eye Health, London School of Hygiene and Tropical Medicine, Keppel Street, London W1E 7HT, UK.; Senior Research Associate (Geriatric Medicine), Department of Medicine, All India Institute of Medical Sciences, New Delhi 110 029, India.

**Figure:**
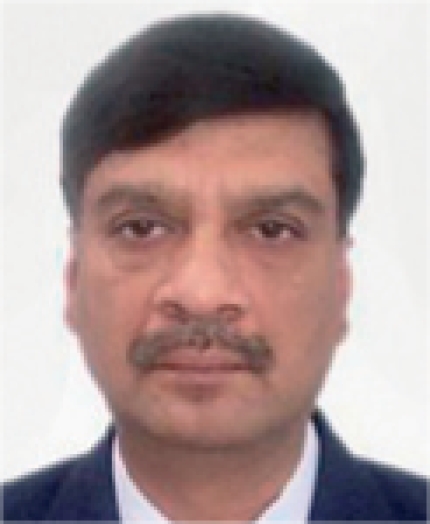


**Figure:**
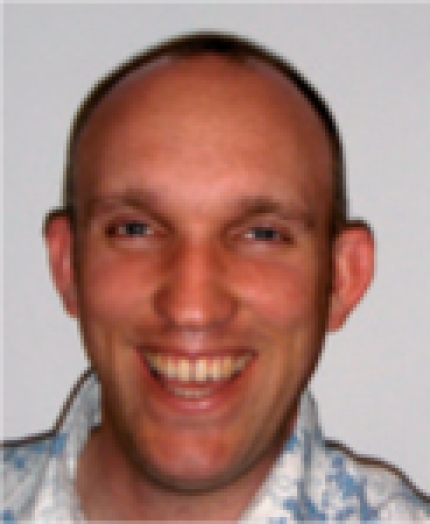


**Figure:**
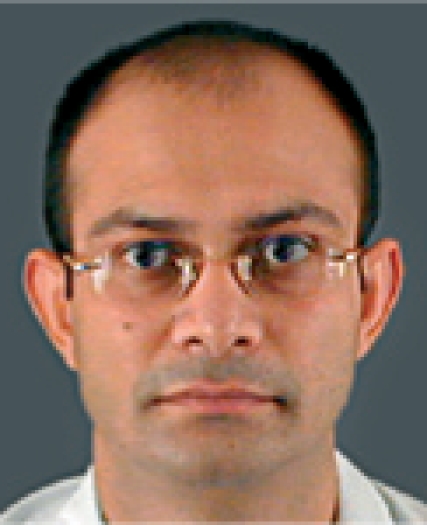


Many eye diseases, such as cataract and age-related macular degeneration, occur more frequently as we advance in years. As a consequence, eye care workers are likely to encounter older people more frequently than any other group.

The holistic approach to treating an older person involves considering the complete person, both physically and psychologically. You should consider every aspect of that person that has an impact on their health and wellbeing. This will greatly improve the outcome of the consultation and any subsequent treatment, both for the older patient and for the eye care worker.

It is equally important to treat every older person who seeks care with respect, in a way that preserves his or her dignity and autonomy.

The following factors should be considered when working with older people; they are discussed in detail below:

- communication
- dependence and decisions
- other health conditions (including drug interactions)
- compliance
- the need for rehabilitation

## Communication

Many older people are affected by hearing and/or cognitive impairment (such as mild dementia), as well as by visual impairment. This affects their ability to communicate effectively. This is especially true in a health care setting: the environment can be perceived as strange or alien, the language used is often unfamiliar, and there is a lot of background noise and activity.

Language or dialect may also be a problem. For example, rural and migrant older people may not speak or understand the language of the health care provider.

The eye care worker should conduct any interaction with an older person in an environment that facilitates communication, i.e. in a location that allows **good face-to-face interaction** (such as a quiet room), where **background noise is minimal**, and there is **little risk of interruption.** When language or dialect is a problem, it is advisable to arrange ahead of time for someone to act as an **interpreter.** If a patient suffers from dementia, it is important to **remain tolerant** - do not get irritated when you have to repeat instructions or explanations.

**Figure:**
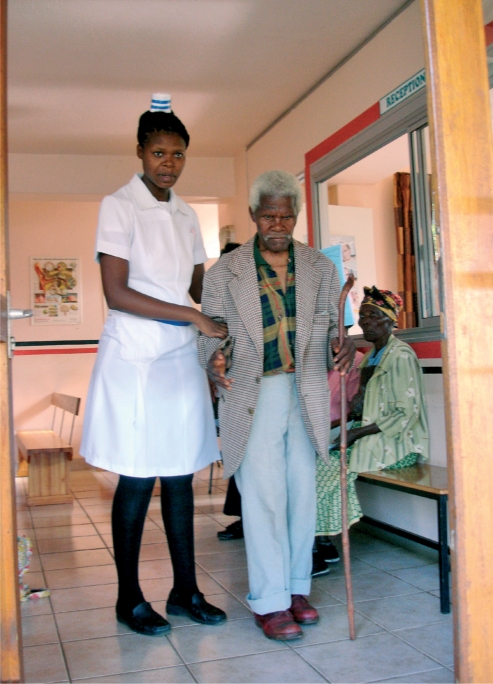
**A nurse assists an older patient in an eye clinic. SWAZILAND**

You should always ensure that any important messages have been received and understood. Use **simple, clear words** instead of medical jargon and, where possible, use relevant leaflets, drawings, photographs, or literature to support your explanations. Giving the older person **written information** to take away will enable them to explain their condition to their family. When patients are illiterate, or functionally illiterate, try to ensure that a family member or friend can assist them with any reading material that is for their own use (such as guidelines for taking medication).

While it is important that you communicate effectively to overcome any difficulties, you must **avoid being patronizing** or ‘talking down’ to an older patient. You should aim to communicate at a level that correctly appreciates the person's situation. To achieve this, there is a need for all staff, medical and support (including receptionists, in particular) to receive special training that will encourage the development of empathy towards older people and appropriate communication skills.

## Dependence and decisions

Older people are often financially dependent on their relatives. Even those who are usually independent may have to ask family members to help them pay for treatment or to contribute to the cost of travelling to an eye care facility.

In addition, older persons may also be physically dependent on a friend or family member to accompany them; this may also cost money and time as it takes the carer or chaperone away from work or childcare responsibilities.

It therefore often happens that older people cannot make decisions about eye care treatment alone - their family will have to be part of the decision-making process.

Eye care practitioners should help patients and their families to make good decisions by:

- talking to them about the diagnosis and treatment options, including side effects of drugs, complications of surgery, and cost
- discussing any additional costs (such as transportation) and what support the patient may need from his or her family
- informing the patient and family members about any other help or support that may be available.

In the case of very complicated technological issues, it may not be possible to make things clear beyond a certain point. In our experience, the family often asks for the doctor's opinion and may empower the doctor to take a surrogate decision. This should be discouraged at all costs because it can be thought to diminish a patient's autonomy. However, in certain cultural settings, it may become unavoidable for the doctor to express an opinion; this is often the case in India, for example. When this happens, doctors can approach the situation by saying: “If I were in your place, I would …”

### Difficult decisions

When difficult decisions need to be made, for example about whether a seriously ill older patient will benefit from cataract surgery, we would suggest the following course of action: **first discuss the situation with the patient on his/her own**, in confidence, in an informal and non-intimidating atmosphere. You can then discuss the same situation in the presence of the family members if the patient so wishes. This will ensure that patients' individual wishes are given preference.

## Other health conditions

Older people are more likely to have multi-organ diseases requiring multiple medications. When planning eye treatment, it is important to be aware of and **understand the impact of these diseases on the individual.** The issues specific to older persons can range from considering drug interactions to finding a way to perform a cataract operation on someone who is unable to lie flat due to shortness of breath.

**Physical disability** may prevent the patient from putting in their own eye drops, from opening bottles and boxes (for example, due to arthritis in the hands), or from walking even a short distance (i.e. from home to the bus).

It is only when the other health needs of older persons have been considered on an individual basis that the person can receive the best care from the eye care provider. Effective two-way communication, both speaking and listening, is vital for this to occur.

### Drug interactions

Adverse drug reactions or interactions are more common in older people. There are two main reasons for this: older people are more susceptible to drugs in general and they often need a greater number of drugs than younger people.

The following are examples of how ocular and systemic medication can adversely affect the older person:

- **Drugs that are administered topically can have local effects:** topical steroids can lead to the development of herpetic corneal ulcers, mydriatics can cause angle-closure glaucoma, and many drops can trigger allergic reactions in patients, often due to the preservatives they contain.
- **Drugs that are administered topically can have systemic effects:** topical beta-blockers can lead to asthma and make obstructive airways disease worse, mydriatics can provoke angle-closure and lead to urinary retention, and pilocarpine (a cholinergic agonist) can cause headaches.
- **Drugs that are administered systemically can affect the eye:** high-dose antimalarials (such as chloroquine) can cause retinopathy, antituberculous drugs (in particular ethambutol) can cause optic neuropathy, and chronic use of oral steroids can lead to cataract.
- **Certain drugs can adversely affect the outcome of ophthalmic surgical procedures:** these include warfarin (risk of bleeding), alpha-antagonists (risk of intraoperative floppy iris syndrome), and topical latanoprost (risk of cystoid macular oedema).

### What should you do?

- Ensure that you have a complete and up-to-date list of the medications the patient is taking.
- Inform patients of possible side effects and their warning signs; advise them to report back early if any such effect is noticed.
- Advise patients to avoid self-medicating.
- Schedule eye check-ups at regular intervals to ensure early detection and treatment.
- Withdraw the offending drug as early as it is safe to do so.

## Compliance

When deciding on a course of treatment, you should also consider the patient's ability to comply with treatment and follow-up.

In the authors' experience, older patients are usually compliant when it comes to matters related to eye disease and vision. However, the importance of regular follow-up and of compliance needs to be reinforced at each visit; written materials, in the form of pamphlets and hand-outs, usually help in this regard. These materials can also help when the support of family members or carers must be enlisted.

## The need for rehabilitation

Despite all measures, a substantial number of older patients finally end up with severe visual disability. For the patient to maintain autonomy and independence in the course of his or her daily activities, it is necessary to organise low vision rehabilitation by a team of professionals, including ophthalmologists, occupational therapists, optometrists, and social workers (see article on page 28). A comprehensive programme of rehabilitation will improve not only quality of vision, but also quality of life for the disabled person.

**Tips for working with older patients**

**Plan ahead**

Older people require more time and patience from the eye care practitioner. The following two suggestions will help you make the best use of your time when caring for these patients:

1. Ask patients to bring with them a list of their medical conditions and drug treatments - this will make the consultation run more smoothly and save time.
2. Volunteers in the clinic can keep older people informed and help them find their way around. These volunteers can also help older people prepare for the consultation and make sure that they are calm when they arrive in the consultation room - this saves time for the nurse or ophthalmologist.

**When you see the patient**

1. Ensure that the environment in which the consultation occurs makes communication as easy as possible.
2. Focus on effective communication. **Remember:** what matters is what the patient understands, not what you say.

**After a diagnosis has been made**

1. Note the following:
  - all current active medical conditions
  - all medications being used (check for any interactions)
  - past adverse drug reactions
  - past medical conditions which may affect the person's eye disease and its treatment, as well as medical conditions which may be affected by it.
2. Describe the different treatment options to the patient. Consider and discuss:
  - the cost of the treatment, if this is likely to be a problem
  - what results the patient can expect
  - any possible side effects of treatment, including discomfort
  - the duration of treatment
  - the amount of help and support the patient may need from family or carers during treatment and what follow-up may be necessary.
3. Support decision-making by the patient and his/her family by making sure you do not hurry them through the consultation.
4. Provide the patient and the family with appropriate information in the form of hand-outs and leaflets. This will optimise compliance and follow-up.

